# Severity of Hypoxia-Induced Effects on 3T3-L1 Adipocyte Secretory Function Is Attenuated Dose-Dependently by Individual Short-Chain Fatty Acids

**DOI:** 10.3390/nu18060942

**Published:** 2026-03-17

**Authors:** Jessie L. Burns, Kelsey Van, Ala Alzubi, Clara E. Cho, Jennifer M. Monk

**Affiliations:** Department of Human Health Sciences, University of Guelph, Guelph, ON N1G 2W1, Canada

**Keywords:** short-chain fatty acids, acetate, propionate, butyrate, adipocytes, inflammation, adipokines, hypoxia, lipopolysaccharide, insulin-stimulated glucose uptake

## Abstract

**Background**: Microbial fermentation of non-digestible carbohydrates and proteins produce short-chain fatty acids (SCFAs), which are critical communication signals in the gut–adipose tissue axis. Individual SCFA can differentially modulate the adipocyte secretory profile and adipose tissue metabolic function; however, their dose-dependent effects on adipocyte function in combined inflammatory and hypoxic environmental conditions that reflect the obesity-associated adipose tissue phenotype remain unknown. **Methods**: Mature 3T3-L1 adipocytes were cultured for 24 h with lipopolysaccharide (LPS; 10 ng/mL) plus 100 µM of cobalt chloride (CoCl_2_) to chemically induce hypoxia ± individual SCFAs, namely acetate (Ace), propionate (Pro), and butyrate (But), in a dose-dependent manner (0.25 mM, 0.5 mM, and 1 mM). **Results**: Ace, Pro and But reduced secretion of IL-6, MCP-1/CCL7 and Rantes/CCL5 in a dose-dependent manner, whereas Pro and But reduced MCP3/CCL7 secretion and only But reduced resistin and increased adiponectin secretion compared to control (*p* < 0.05). Intracellular protein expression of the ratio of phosphorylated–to–total NFκB p65 was reduced by 1 mM But, whereas the ratio of phosphorylated–to–total STAT3 expression was reduced by 1 mM Ace, Pro and But and 0.5 mM Pro and But compared to control (*p* < 0.05). There was no difference in insulin-stimulated or non-insulin-stimulated glucose uptake between control and any individual SCFAs (*p* > 0.05). **Conclusions**: Adipocyte adipokine secretory function in combined inflammation and hypoxic environmental conditions is dose-dependently attenuated by individual SCFA, which exhibit both individual and overlapping effects.

## 1. Introduction

In obese adipose tissue, chronic low-grade inflammation, also referred to as metainflammation, has been shown to promote metabolic dysfunction, wherein the secretion of adipose tissue-derived inflammatory adipokines (i.e., cytokines, chemokines and hormones) into the systemic circulation further perpetuates dyslipidemia and insulin resistance [1,2,3,4,5]. As adipose tissue expands in size, adipocytes can become hypoxic, and the increased production of adipokines in the combined inflammatory and hypoxic adipose tissue microenvironment increases the severity of metabolic dysfunction compared to normoxic conditions [3,6,7,8,9,10]. Additionally, the changes in the gastrointestinal microbiome composition and function in obesity [5,11] can influence the gut–adipose tissue axis [12]. Specifically, various microbial metabolites (including but not limited to short-chain fatty acids (SCFAs), branched-chain fatty acids, secondary bile acids, and trimethylamine *N*-oxide) that are produced from undigested dietary precursors become critical communication signals between the gastrointestinal tract (microbiome and host epithelial barrier combined) and the adipose tissue [12]. Amongst these bioactive microbial metabolites, SCFAs are of interest based on their ability to influence both gastrointestinal and adipose tissue function. Specifically, SCFAs have been shown to influence intestinal epithelial barrier integrity (via their influence on tight junctions) and mucosal integrity [13] and stimulate secretion of gut hormones [e.g., glucagon-like peptide-1 (GLP-1) and peptide YY (PYY)] and neurotransmitters including gamma-aminobutyric acid (GABA) and 5-hydroxytryptamine (5-HT) [12,14,15]. Moreover, SCFAs have been shown to modulate metabolic function not only in adipose tissue but also in skeletal muscle, liver and pancreas [14,16,17,18,19,20], and therefore not only influence the gut–adipose tissue axis but can exert systemic effects that can attenuate the severity of the obese phenotype. As proof-of-concept, intervention studies have been conducted wherein increased production of SCFAs from non-digestible carbohydrates have been shown to attenuate obesity severity and improve metabolic function [21,22,23,24,25,26,27,28,29,30,31,32,33,34,35,36,37,38,39,40,41,42,43]. Thus, increasing circulating SCFA levels represents a potential intervention strategy to limit the severity of obesity-associated adipose tissue dysfunction.

SCFAs are produced from microbial fermentation of non-digestible or microbially accessible carbohydrates [44,45], and to a lesser degree, can also be produced from the fermentation of undigested protein [16,46,47,48,49]. Between 90 and 95% of colonic SCFAs are in the form of acetate (or acetic acid), propionate (or propionic acid), and butyrate (butyric acid), typically produced in a 3:1:1 molar ratio [50,51,52,53,54], although SCFAs with longer carbon chains can also be produced in lower amounts [55]. Circulating levels of SCFAs are lower than fecal or gastrointestinal luminal concentrations [16,17,18,19,20] and their production can vary between individuals based on multiple factors including dietary intakes of non-digestible carbohydrates, microbiome composition and intestinal SCFA absorptive capacity [16,56,57,58,59,60,61]. Therefore, there is variability in circulating levels of SCFAs, which highlights the importance of determining the optimal SCFA concentration required to influence adipose tissue function. SCFAs exert their effects mechanistically though G protein-coupled receptors (GPR; mainly GPR41, GPR43, and GPR109a), wherein individual SCFAs exert different binding affinities for each receptor type [62,63,64]. Additionally, butyrate has been shown to function as a histone deacetylase inhibitor [65], which can affect the epigenome through chromatin remodeling changes. Importantly, SCFAs are presumed to exert the same biological effects; however, we have shown that individual SCFAs exert differential effects on the secretory profile of inflammatory mediators from both skeletal muscle cells (i.e., myokine secretion) [66,67] and adipocytes (i.e., adipokine secretion) [68,69]. Importantly, the effects of individual SCFAs (e.g., acetate, propionate and butyrate) on the adipokine secretory profile in normoxic environmental conditions were shown to be dose-dependent, with the most significant effects attributed to butyrate, which resulted in both an anti-inflammatory and anti-chemotactic adipokine secretory profile [68]. These findings highlight the relevance of ascertaining the effective dose and individual SCFAs that can beneficially modulate obese adipocyte function (both inflammatory mediator secretion and/or metabolic function) to better target the gut–adipose tissue axis.

Cell culture models can provide a valuable experimental approach to evaluate the effects of individual SCFAs on adipocyte function. Identifying the concentration and individual SCFAs that can beneficially modulate adipocyte function can inform intervention strategies (prebiotic or supplemental approaches) that would result in the optimal SCFA concentrations in the blood to modulate the gut–adipose tissue axis. Moreover, appropriately crafted cell culture models can recapitulate the critical features of the more severe obese adipose tissue cellular microenvironment, such as the combined inflammatory stimulus of endotoxemia (increased circulating lipopolysaccharide) and hypoxia, which exacerbates adipokine secretion and metabolic dysfunction [3,6,7,8,9,10,70,71]. Therefore, utilizing a SCFA concentration range that both includes and extends beyond circulating levels reported in humans [72,73,74,75,76] into supraphysiologic concentrations that could be achieved via encapsulated SCFA supplements [77], this study determined the dose-dependent effects of individual SCFAs on the adipocyte adipokine secretory profile and metabolic function in combined inflammatory and hypoxic environmental conditions.

## 2. Materials and Methods

### 2.1. 3T3-L1 Cell Culture, Differentiation and Hypoxia Stimulation Conditions

3T3-L1 murine pre-adipocytes (CL-173; American Type Culture Collection, Manassas, VA, USA) were cultured and maintained according to the manufacturer’s instructions in Dulbecco’s modified Eagle’s medium (DMEM; HyClone, Logan, UT, USA) supplemented with 4 mM L-glutamine, 4500 mg/L glucose, 10% (*v*/*v*) low endotoxin sterile-filtered fetal bovine serum (FBS; Millipore-Sigma, Oakville, ON, Canada) and 1% (*v*/*v*) penicillin-streptomycin (Fisher Scientific, Mississauga, ON, Canada), as described previously [68,69,78]. Differentiation of pre-adipocytes into lipid-laden adipocytes was performed using DMEM supplemented with 1 µmol/L dexamethasone, 0.5 mM 3-isobutyl-1-methylxanthine, and 10 µg/mL insulin (all from Millipore-Sigma), and subsequently cells were matured in DMEM supplemented with 10 µg/mL insulin, as described previously [68,69,78]. Media were changed every two days.

On day 8 post-differentiation, adipocytes were incubated for 12 h in serum-free DMEM containing 1% (*v*/*v*) penicillin-streptomycin prior to the addition of the experimental treatments. Environmental hypoxia was induced by the addition of 100 µM of the hypoxia-mimetic compound, cobalt chloride (CoCl_2_; #15862; Millipore-Sigma), which has been shown to mimic the effects of low (1%) oxygen tension between adipocytes over 24 h without affecting cell viability, as we and others have shown previously [69,79,80]. Furthermore, comparison between hypoxia methodological approaches, namely gas-based and CoCl_2_, have shown no difference in the degree of cell apoptosis [81]. In confirmation, cell viability in response to all treatment conditions was assessed using Trypan blue exclusion and exceeded 90%. Normoxic negative control cultures (i.e., without CoCl_2_; *n* = 8) were included. A combined inflammatory hypoxic environmental condition has been shown to recapitulate the metabolic dysfunction associated with obese adipocytes [69,80], consisting of 100 µM CoCl_2_ plus 10 ng/mL lipopolysaccharide [LPS, from *Escherichia coli* 055:B5 (#L5418; Millipore-Sigma)]. Therefore, 3T3-L1 adipocytes treated with the combined inflammatory hypoxic environmental conditions served as the positive control (CON + LPS/CoCl_2_; *n* = 8). The concentration of LPS utilized in these experiments recapitulates the circulating endotoxin levels identified in humans with obesity and in rodent obesity models [82,83,84]. In both the hypoxic (+CoCl_2_) and the combined inflammatory hypoxic (+LPS/CoCl_2_) environmental conditions, adipocyte cultures were treated for 24 h dose-dependently (0.25 mM, 0.5 mM, or 1 mM) with individual SCFAs, namely sodium acetate (Ace), sodium propionate (Pro), and sodium butyrate (But) (all from Millipore-Sigma; *n* = 8–9/group). The dose-dependent effect of individual SCFAs in normoxic environmental conditions has been reported elsewhere [68]. After 24 h, culture supernatant was collected and stored at −80 °C to await secreted adipokine analysis. Adipocyte cell lysates were collected and cellular protein was isolated using the RNA/Protein Purification Plus Kit (Norgen Biotek Corp., Thorold, ON, Canada), and stored at −80 °C.

### 2.2. Intracellular Protein Analysis

Total intracellular protein was quantified using the bicinchoninic assay and an equal amount of protein (10 µg/sample/assay) was used to measure the ratio of phosphorylated-to-total expression of STAT3 (phosphorylated STAT3 [Tyr705]–total STAT3) and NFκB p65 (phosphorylated NFκB p65 [Ser536]–total NFκB p65) using an enzyme-linked immunosorbent assay (Thermo-Fisher Scientific, Mississauga, ON, Canada), as described previously [68,69].

### 2.3. Secreted Adipokines

Adipocyte secretion cytokines (IL-1β, IL-6 and TNFα), chemokines (RANTES/CCL5, MCP-1/CCL2, MCP-3/CCL7, MIP-1α/CCL3 and MIP-1β/CCL4) and hormones (adiponectin, leptin and resistin) were measured by multiplex using the Bio-Plex 200 system and accompanying Plex Manager software, version 6.0 (Bio-Rad, Mississauga, ON, Canada), as described [68,69].

### 2.4. Glucose Uptake Assay

Mature 3T3-L1 adipocyte cultures treated with LPS/CoCl_2_ and either CON or 1 mM Ace, Pro, or But for 24 h (*n* = 9/treatment group) were used to assess glucose uptake under either basal (i.e., non-insulin stimulated: phosphate-buffered saline for 20 min followed by 10 mM 2-deoxy glucose for 20 min at 37 °C) and insulin-stimulated (1 µM insulin for 20 min followed by 10 mM 2-deoxy glucose for 20 min at 37 °C) using the colorimetric Glucose Uptake Assay Kit (Abcam, Waltham, MA, USA) according to the manufacturer’s instructions and as described previously [66,68]. Negative control glucose-starved cell cultures [*n* = 4/treatment group (Con, Ace, Pro and But)] that did not receive either 2-deoxy glucose or insulin served as background negative control cultures. There was no difference in optical density between the control and SCFA-negative control cultures (as seen previously [68]).

### 2.5. Statistical Analysis

All data are expressed as means ± SEM. Data were analyzed by one-way ANOVA followed by Tukey’s multiple comparisons test for post hoc analysis between experimental treatment groups (*p* ≤ 0.05). The Shapiro–Wilk test was used to test for normality. All analyses were conducted using GraphPad Prism, version 10 (GraphPad Software, Inc., La Jolla, CA, USA).

## 3. Results

### 3.1. Dose-Dependent Effect of Individual SCFAs on Adipokine Secretion in a Hypoxic Environment

**Figure 1:**
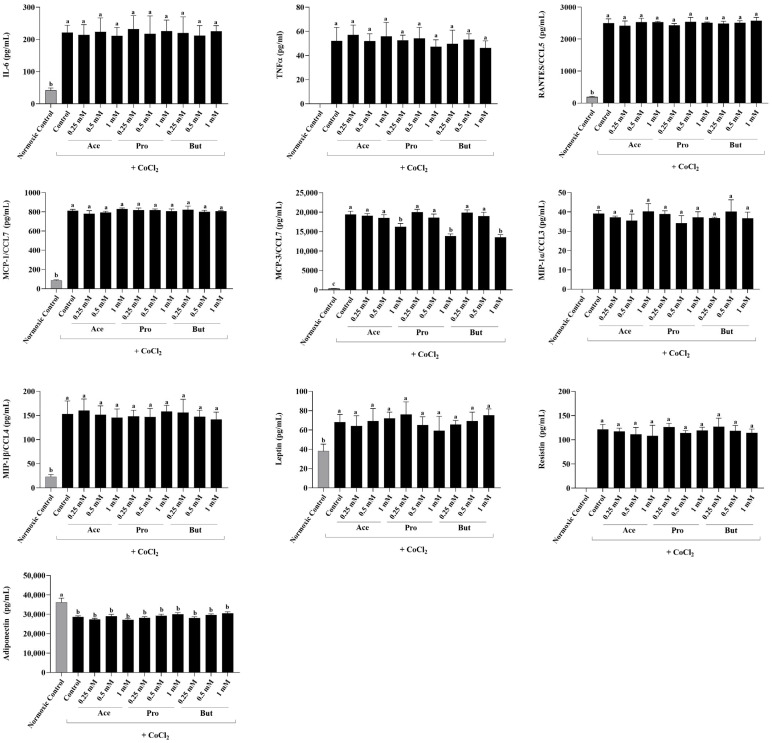
Secreted adipokine levels from 3T3-L1 adipocytes in response to the dose-dependent addition of individual SCFAs (Ace, Pro and But at 0.25 mM, 0.5 mM and 1 mM) in hypoxic environmental conditions (10 µM CoCl_2_; black bars). Normoxic control cultures (without CoCl_2_) served as negative controls (gray bars). Bars represent mean values ± SEM (*n* = 8–9/experimental group). Data were analyzed by one-way ANOVA followed by Tukey’s multiple comparison test. Bars not sharing a lower-case letter differ (*p* < 0.05).

The dose-dependent effect of individual SCFAs (Ace, Pro and But) on adipokine secretion in hypoxic environmental conditions compared to both a hypoxic control and a normoxic negative control is shown in Figure 1. As shown previously [69], when compared to the normoxic negative control, the secretion of all adipokines were increased in the hypoxic environmental conditions, with the exception of adiponectin, whose secretion was decreased in hypoxia (*p* < 0.05). TNFα, MIP-1α/CCL3 and resistin secretion were not detectable in the normoxic negative control group. Additionally, IL-1β secretion was below the limit of detection in both the normoxic negative control group and all experimental groups in hypoxic environmental conditions (Control and each individual SCFA). In response to environmental hypoxia induced by CoCl_2_, only the secretion of MCP-3/CCL7 was dose-dependently decreased by the presence of a 1 mM concentration of Ace, Pro and But (*p* < 0.05), whereas each SCFA had no effect on MCP-3/CCL7 secretion at the lower 0.5 mM and 0.25 mM SCFA concentrations. There was no effect of each SCFA at any concentration assessed on the secretion of any other adipokines in the hypoxic environmental condition compared to the hypoxic control group (*p* > 0.05).

### 3.2. The Adipocyte Adipokine Secretory Profile Is Dose-Dependently Reduced by SCFAs in an Inflammatory and Hypoxic Environment

**Figure 2:**
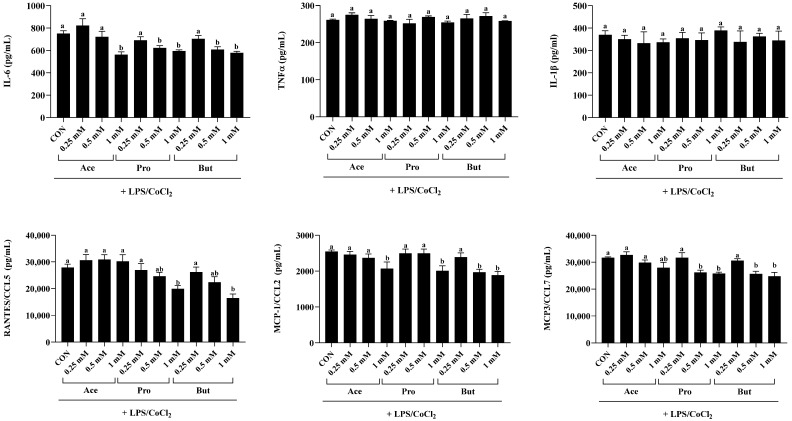

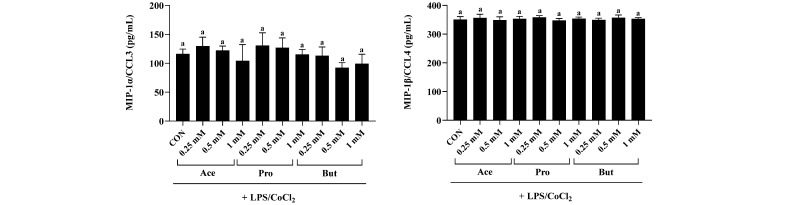
Secreted adipokine levels (i.e., cytokines and chemokines) from 3T3-L1 adipocytes in response to the dose-dependent [0.25 mM, 0.5 mM and 1 mM] addition of individual SCFAs (Ace, Pro and But) in combined inflammatory and hypoxic environmental conditions (+LPS/CoCl_2_). Bars represent mean values ± SEM (*n* = 8–9/experimental group). Data were analyzed by one-way ANOVA followed by Tukey’s multiple comparison test. Bars not sharing a lower-case letter differ (*p* < 0.05).

Secretion of cytokines and chemokines from adipocytes stimulated with combined inflammatory and hypoxic environmental conditions was dose-dependently decreased by SCFAs, as shown in Figure 2. As expected, adipokine secretion was significantly lower in the hypoxic condition (CoCl_2_ alone; Figure 1) compared to the inflammatory hypoxic (LPS + CoCl_2_; Figure 2) condition (*p* < 0.05). SCFAs had a dose-dependent effect on reducing IL-6 secretion within inflammatory hypoxic environmental conditions compared to CON (*p* < 0.05; Figure 2). Specifically, the 1 mM concentration of Ace, Pro and But reduced IL-6 secretion versus CON (*p* < 0.05), but did not differ from each other. At the 0.5 mM concentration, only Pro and But reduced IL-6 secretion compared to CON *(p* < 0.05) and did not differ from each other. There was no difference between experimental groups in IL-6 secretion at the 0.25 mM concentration (*p* > 0.05). Conversely, there was no difference in TNFα or IL-1β secretion between CON and any SCFA at any concentration assessed (*p* > 0.05; Figure 2).

With respect to chemokine secretion, RANTES/CCL5 was reduced by only the 1 mM concentration of Pro and But compared to CON (*p* < 0.05), whereas the 1 mM concentration of Ace did not differ from CON (*p* > 0.05). The 0.5 mM and 0.25 mM concentrations of each SCFA had no effect on RANTES/CCL5 secretion. Only the 1 mM dose of Ace, Pro and But reduced secretion of MCP-1/CCL2 compared to CON (*p* < 0.05), and did not differ from each other. Additionally, only the 0.5 mM concentration of But reduced MCP-1/CCL2 secretion compared to CON (*p* < 0.05), whereas the 0.5 mM concentration of Ace and Pro and all three SCFAs at the 0.25 mM concentration did not differ from CON (*p* > 0.05). MCP3/CCL7 secretion was reduced by both the 1 mM and 0.5 mM concentrations of Pro and But compared to CON (*p* < 0.05), whereas the 0.25 mM concentration of Pro and But, along with all Ace concentrations (1, 0.5 and 0.25 mM), did not differ from CON or each other (*p* > 0.05). There was no difference between experimental groups in the secretion of MIP-1α/CCL3 or MIP-1β/CCL4 (*p* > 0.05).

**Figure 3:**
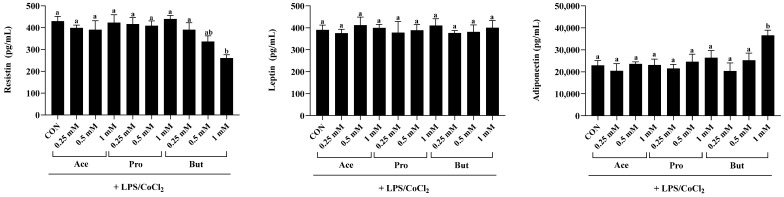
Secreted adipokine levels (i.e., resistin, leptin and adiponectin) from 3T3-L1 adipocytes in response to the dose-dependent [0.25 mM, 0.5 mM and 1 mM] addition of individual SCFAs (Ace, Pro and But) in combined inflammatory and hypoxic environmental conditions (+LPS/CoCl_2_). Bars represent mean values ± SEM (*n* = 8–9/experimental group). Data were analyzed by one-way ANOVA followed by Tukey’s multiple comparison test. Bars not sharing a lower-case letter differ (*p* < 0.05).

Secreted levels of the inflammatory adipokine resistin were significantly reduced only by But at the 1 mM concentration compared to CON (*p* < 0.05; Figure 3). At the same 1 mM concentration, there was no difference in secreted resistin levels between CON, Ace and Pro (*p* > 0.05); however, at the 1 mM concentration Ace and Pro differed from But (*p* < 0.05). At the lower SCFA concentrations (0.5 mM and 0.25 mM), there was no difference in secreted resistin levels between any SCFA or CON (*p* > 0.05). There was no difference in leptin secretion levels between CON and any SCFA at any of the concentrations assessed (*p* < 0.05). Conversely, compared to CON the 1 mM concentration of But significantly increased (*p* < 0.05) secreted levels of adiponectin. In contrast, secreted levels of adiponectin did not differ between the 1 mM concentration of Ace and Pro versus CON (*p* < 0.05). However, adiponectin secretion from adipocytes treated with 1 mM Ace and Pro were significantly lower compared to But (*p* < 0.05). There was no difference in adiponectin secretion between CON and any individual SCFA at the lower 0.25 mM and 0.5 mM concentrations (*p* > 0.05).

### 3.3. Adipocyte Transcription Factor Activation Status (Ratio of Phosphorylated to Total) Is Dose-Dependently Reduced by SCFAs in an Inflammatory and Hypoxic Environment

**Figure 4:**
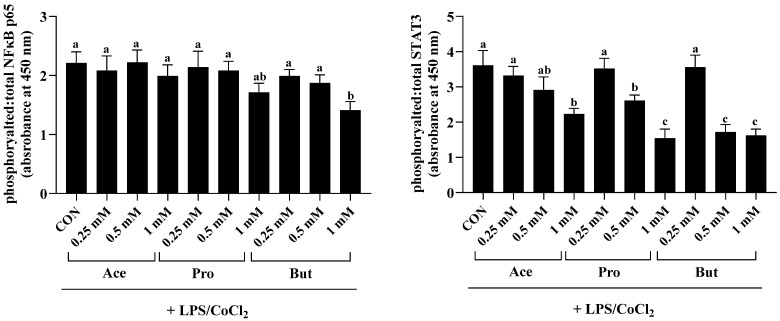
3T3-L1 adipocyte intracellular protein expression of the ratio of phosphorylated to total NFκB p65 and STAT3 in response to the dose-dependent [0.25 mM, 0.5 mM and 1 mM] addition of individual SCFAs (Ace, Pro and But) in combined inflammatory and hypoxic environmental conditions (+LPS/CoCl_2_). Bars represent mean values ± SEM (*n* = 8–9/experimental group). Data were analyzed by one-way ANOVA followed by Tukey’s multiple comparison test. Bars not sharing a lower-case letter differ (*p* < 0.05).

The activation status, namely the ratio of phosphorylated to total NFκB p65, was reduced only by the 1 mM dose of But compared to CON (*p* < 0.05; Figure 4). The lower concentration of But (0.5 and 0.25 mM) did not differ from CON (*p* > 0.05). Further, there was no difference in the intracellular levels of phosphorylated to total NFκB p65 between CON and either Ace or Pro at any concentration tested (*p* > 0.05). Conversely, the ratio of phosphorylated to total STAT3 was significantly reduced by the 1 mM dose of Ace, Pro and But compared to CON (*p* < 0.05), wherein Pro and But were significantly lower compared to Ace. Additionally, at the 0.5 mM dose, But and Pro reduced STAT3 activation status compared to CON, wherein But was significantly lower compared to Pro (*p* < 0.05). Both the 0.5 mM and 0.25 mM doses of Ace did not differ from CON, and similarly, the 0.25 mM dose of Pro and But did not differ from CON (*p* > 0.05).

### 3.4. Individual SCFAs Do Not Affect Insulin-Stimulated Glucose Uptake in 3T3-L1 Adipocytes in Combined Inflammatory and Hypoxic Environmental Conditions

**Figure 5:**
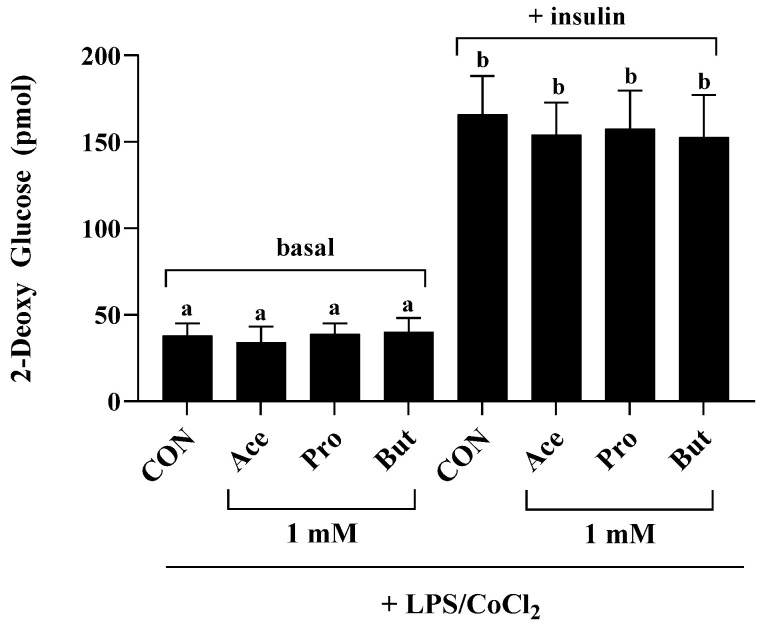
2-dexoy glucose uptake by 3T3-L1 adipocytes treated with 1 mM Ace, Pro or But under combined inflammatory and hypoxic environmental conditions (+LPS/CoCl_2_) in basal (i.e., non-insulin stimulated: phosphate-buffered saline for 20 min followed by 10 mM 2-deoxy glucose for 20 min at 37 °C) and insulin-stimulated (1 µM insulin for 20 min followed by 10 mM 2-deoxy glucose for 20 min at 37 °C) conditions (*n* = 8–9/treatment group: CON, Ace, Pro and But in either basal or insulin-stimulated condition). Bars represent mean values ± SEM. Data were analyzed by one-way ANOVA followed by Tukey’s multiple comparison test. Bars not sharing a lower-case letter differ (*p* < 0.05).

Glucose uptake in both basal (i.e., non-insulin-stimulated) and insulin-stimulated conditions (Figure 5), was assessed only at the 1 mM SCFA concentration + LPS/CoCl_2_ experimental conditions, which were the experimental conditions that most significantly affected the adipocyte secretory profile (Figure 2 and Figure 3). Basal glucose uptake did not differ between experimental groups (*p* > 0.05); however, as expected, glucose-uptake was increased under the insulin-stimulated condition compared to basal glucose uptake levels in all treatment groups (CON, Ace, Pro and But) (*p* < 0.05). There was no difference between CON and any SCFA treatment group in insulin-stimulated glucose uptake (*p* > 0.05).

## 4. Discussion

The current study assessed the dose-dependent effects of individual SCFAs (Ace, Pro and But) on the adipocyte adipokine secretory profile and glucose-uptake capability within a combined inflammatory and hypoxic cellular environment. In obesity, hypoxic adipose tissue, compared to normoxic conditions, has been shown to have a more aggressive inflammatory adipokine secretory profile that drives metabolic dysfunction including dyslipidemia and insulin resistance [1,2,3,4,5,6,7,8,9,10,85]. The influence of individual SCFAs on the adipokine secretory profile represents a critical intervention point to attenuate the severity of obesity-associated adipocyte dysfunction, and therefore, SCFAs represent critical communication signals in the gut–adipose tissue axis [12]. Dietary interventions with non-digestible carbohydrates (i.e., SCFA precursors) have been shown to attenuate the severity of the obese phenotype, including reducing weight gain [21,22,23,24,25,26,30,36,37,38,39,40,41,42,43,86], reducing adipose tissue mass [21,22,23,24,25,26,27,28,29], and improving metabolic outcomes, including dyslipidemia, blood glucose regulation, and/or insulin resistance [25,26,30,31,32,33,34,35,87].

To recapitulate obesity-associated inflammatory and hypoxic adipose tissue environmental conditions [3,6,7,8,9,10,70,71], the dose of LPS utilized reflects the level of circulating endotoxin in both rodent models and humans with obesity [82,83,84]. Furthermore, the induction of hypoxia using CoCl_2_ had been used previously to mimic low (i.e., 1%) oxygen tension between adipocytes over the 24 experimental period [69,79,80]. In addition, the dose of SCFA utilized herein has been used previously [68,69,88,89,90,91,92,93], and at the lower concentrations utilized reflects circulating levels reported in humans [72,73,74,75,76]. Other studies have demonstrated the metabolic effects of SCFAs [89,94,95,96,97,98] using concentrations that may not be physiologically attainable through dietary interventions (via increased intakes non-digestible carbohydrates), and thus may not reflect the subsequent SCFA concentrations that adipose tissue would be likely to encounter in vivo. In this connection, the concentration range evaluated in the current dose–response study utilized a concentration range that both reflects circulating SCFA levels reported in humans [72,73,74,75,76] and supraphysiological concentrations that are higher than what is achievable in vivo from fermentation of non-digestible dietary precursors. Importantly, hydroxypropyl methylcellulose-encapsulated SCFAs have been shown to be an effective method to increase circulating concentrations of SCFAs [77] and could be used either alone or as an adjunct to dietary prebiotic and/or probiotic interventions to increase circulating SCFA levels, including to concentrations identified herein that exerted the strongest biological effects. Therefore, it remains important to evaluate the effects of individual SCFAs and determine the dose of each SCFA required to exert beneficial effects on adipocyte function (and within other tissues), particularly since SCFA gastrointestinal production levels, and by extension but at a lower concentration, circulating SCFA levels, will vary between individuals. This variability in SCFA production is based on multiple factors including (i) the microbiome composition (i.e., abundance of SCFA-producing microbial species); (ii) the amount and type(s) of non-digestible carbohydrates consumed in the diet (i.e., SCFA precursors such as, but not limited to, soluble fiber, resistant starches, oligosaccharides, etc.); (iii) the relative fermentability of each type of non-digestible carbohydrate consumed (i.e., fast versus slowly fermentable); and (iv) the intestinal SCFA absorptive capacity [16,56,57,58,59,60,61]. Thus, optimized concentrations of encapsulated SCFAs could represent a precise strategy to enhance the efficacy of prebiotic and/or probiotic interventions to tailor SCFA concentrations at target tissues to achieve the necessary levels required to exert beneficial effects.

Few studies have evaluated the effects of individual SCFAs on cytokine and/or chemokine gene or protein expression [68,69], with some utilizing concentrations (3 mM) that adipose tissue may not encounter in vivo and focus only on effects of only propionate [99], or utilized adipocytes in co-culture with macrophages [91]. However, within inflammatory and hypoxic environmental conditions mimicking obese adipose tissue conditions, individual SCFAs exerted both independent and common effects on the adipokine secretory profile, which were dose-dependent. Furthermore, the dose-dependent effects of Ace, Pro and But on the inflammatory and hypoxic adipocyte adipokine secretory profile in the current study were similar to those previously observed under normoxic environmental conditions [68]; however, a dose–response of individual SCFAs under hypoxic environmental conditions has not been evaluated. We have previously identified the effects of butyrate under both normoxic and hypoxic inflammatory conditions in comparison to the 5 carbon SCFA valerate and the comparable carbon chain length branched chain fatty acids iso-butyrate and iso-valerate [69]; however, the current study utilized a dose–response to evaluate more physiologically relevant SCFA concentrations and permitted the comparison to the more commonly produced acetate and propionate. Additionally, the current study extends what is known about the effects of butyrate (along with acetate and propionate) on the secretion of the adipokines leptin, resistin and adiponectin, along with a functional assessment of glucose uptake in inflammatory hypoxic environmental conditions, which was not evaluated previously [69]. Individual SCFAs can exert both individual and overlapping effects and should not be anticipated to act similarly. In the current study, under inflammatory and hypoxic conditions, But most potently attenuated inflammatory and chemotactic adipokine secretion compared to Pro and Ace, which is in contrast to the longer 5 carbon SCFA valerate, which was shown to promote adipocyte inflammatory and chemotactic adipokine secretion in the same conditions [69]. Specifically, the 1 mM concentration in the current study was the most effective, wherein Ace, Pro and But reduced secretion of IL-6, MCP-1/CCL2 and Rantes/CCL5, whereas only Pro and But reduced secretion of MCP3/CCL7. Additionally, both Pro and But reduced secretion of IL-6 and MCP3/CCL7 at the lower 0.5 mM concentration. There were no effects of any SCFA at the lowest (0.25 mM) concentration, which more accurately recapitulate circulating SCFA levels in humans [72,73,74,75,76]. In addition to the aforementioned effects, 1 mM But was the only SCFA that further modified the adipokine secretory profile by also reducing resistin secretion and increasing adiponectin secretion. Furthermore, the activation (i.e., ratio of phosphorylated-to-total) of the transcription factors that influence the expression of the adipokine secretory profile, namely STAT3 and NFκB p65, were reduced by SCFAs. These transcription factors drive an inflammation feedback loop that upregulates the expression of many inflammatory adipokines [100], and therefore, the ability to reduce their activation can mechanistically dampen the adipokine secretory profile. Importantly, all three SCFAs reduced STAT3 activation at the 1 mM concentration, whereas only Pro and But reduced STAT3 activation at the 0.5 mM concentration. Conversely, only the 1 mM concentration of But reduced NFκB p65 activation. Within adipose tissue, IL-6 plays a key inflammatory role in addition to influencing macrophage infiltration, stimulating leptin secretion and influencing metabolic function including lipolysis and acting both locally and systemically to promote insulin resistance and glucose intolerance [101,102,103,104,105,106]. In this connection, leptin secretion did not differ between any SCFAs and CON. Furthermore, secretion of the adipose tissue monocyte chemotactic signals such as MCP1/CCL2 and Rantes/CCL5 (that are elevated in obese adipose tissue), were also reduced by all SCFAs, which function to promote monocyte differentiation into M1 macrophage functional phenotypes and promote insulin resistance [2,3,107,108,109,110,111]. Of note, the influence of both Pro and But on reducing MCP-3/CCL7 highlights the overlapping effects of some SCFAs to antagonize multiple monocyte chemotactic mediators [112], which, combined with the effect of Rantes/CCL5 on T cell adipose tissue accumulation [113], suggests the potential to reduce the immune cell infiltration into adipose tissue that further perpetuates adipose tissue secretion of inflammatory mediators and ongoing metabolic dysfunction [1,2,3,4,5,6,7,8,9,10,85].

Adipocyte-derived hormones represent a critical component of the adipokine secretory profile. Previous studies evaluating the effects of SCFAs on either adipocyte or adipose tissue explants have tended to focus on leptin and adiponectin [89,92,102,114], versus a broader array of adipokines. Leptin secretion was not affected by SCFAs under inflammatory hypoxic environmental conditions in this study; however, under normoxic conditions leptin secretion was decreased by Ace, Pro and But [68], highlighting the differential effects of individual SCFAs under different inflammatory conditions that reflect the severity of obesity-associated adipose tissue inflammation [1,2,3,4,5,6,7,8,9,10,85]. Conversely, resistin secretion was reduced only by the 1 mM concentration of But, which is functionally relevant given that elevated resistin levels in obesity [115,116] perpetuate adipose tissue inflammation by activating NFκB and driving the expression of inflammatory adipokines including MCP-1 and IL-6, amongst others [117]. NFκB p65 activation and secretion of MCP-1/CCL2 and IL-6 were all reduced by But in the current study. Interestingly, only But was shown to increase the secretion of the anti-inflammatory and insulin-sensitizing adipokine, adiponectin [118], whose beneficial effects are attenuated in obesity [119,120].

Previous studies have demonstrated the ability of SCFAs to decrease lipolysis and increase triglyceride accumulation, along with increasing adipogenesis and glucose uptake [80,90,91,92,93,94,121,122,123,124], although the concentrations used in some of these studies exceed circulating SCFA levels reported in humans. Previously, under inflammatory normoxic conditions, we found no difference in adipocyte insulin-stimulated glucose uptake between 1 mM acetate, propionate and butyrate [68], and similarly, in the current study under inflammatory hypoxic environmental conditions there was no difference in adipocyte insulin-stimulated glucose uptake between individual SCFAs (assessed at a 1 mM concentration). Previous studies utilizing unstimulated primary rat adipocytes have shown conflicting findings, wherein a 10 mM dose of propionate and butyrate decreased basal glucose uptake, whereas 1, 3 and 10 mM concentrations of either propionate or butyrate were shown to increase insulin-stimulated glucose uptake [123]. Conversely, in unstimulated 3T3-L1 adipocytes only a 300 µM concentration of propionic acid increased insulin-stimulated glucose uptake, a response that was shown to plateau at higher concentrations including 1 mM [93], the same SCFA concentration utilized in the current study under inflammatory hypoxic environmental conditions. Additional evidence of a SCFA-mediated effect on adipose tissue glucose uptake capabilities comes from omental adipose tissue from subjects with overweight, which exhibited increased *Glut4* mRNA expression following 24 hour incubation with 3 mM propionate [98]. These findings highlight the differences between experimental models (i.e., primary intact tissue, primary adipocytes and cell lines) and the importance of considering the environmental conditions mimicking the in vivo setting of obese adipose tissue, which can influence the effect of SCFAs on insulin-stimulated glucose-uptake. Importantly, the addition of 5% *w*/*w* sodium butyrate to a high-fat diet improved insulin sensitivity, reduced fasting glucose and insulin levels; decreased homeostasis model assessment for insulin resistance (HOMA-IR); and showed an improved response to an intraperitoneal insulin tolerance test [125]. In a separate study, the addition of 1% sodium butyrate to the drinking water in combination with a high-fat diet was shown to reduce fasting glucose and fasting insulin levels, along with an improved response to an intraperitoneal glucose tolerance test [126]. Future studies should conduct a more in-depth evaluation of the effects of individual SCFAs across a range of concentrations on diverse aspects of adipocyte metabolic function, not just insulin-stimulated glucose uptake, under inflammatory hypoxic environmental conditions.

Collectively, the current study demonstrates the dose-dependent effects of the individual SCFAs acetate, propionate and butyrate in combined inflammatory and hypoxic environmental conditions that reflect the severity of hypoxic obesity-associated adipose tissue function [1,2,3,4,5,6,7,8,9,10,85]. Herein, we demonstrated that butyrate had the broadest effect at attenuating the inflammatory and chemotactic adipokine secretory profile in comparison to the shorter carbon chain length SCFAs, with propionate having an intermediate effect and acetate having the weakest effect on the adipokine secretory profile. Importantly, butyrate also concomitantly increased adiponectin secretion, thereby demonstrating its unique ability to promote anti-inflammatory and insulin-sensitizing adipokine secretion, although this did not result in a functional difference in insulin-stimulated glucose uptake capability between individual SCFAs. Furthermore, these results demonstrate the differential effects of individual SCFAs on hypoxic adipocyte secretory function that align with previous studies demonstrating that individual SCFAs do not necessarily exert the same effects on adipocyte function in either normoxic or hypoxic conditions [68,69], or by extension to another tissue, on skeletal muscle function [66,67]. This is despite the shared SCFA-mediated mechanisms of action, including GPR signaling [62,63,64], and the unique mechanism of butyrate to function as a histone deacetylase inhibitor [65]. Future studies should assess the adipocyte GPR expression pattern and utilize signaling inhibitors such as the GPR inhibitor, pertussis toxin, and/or the histone deacetylase inhibitor, trichostatin A, under both normoxic and hypoxic environmental conditions, as conducted in other cell types such as neutrophils [127] or co-cultured adipocytes and macrophages [91]. Additionally, morphologic changes in adipocytes, including changes in lipid accumulation in both normoxic and hypoxic environmental conditions could be assessed, along with comparative studies evaluating the adipocyte response (± SCFA) to gas and chemically induced environmental hypoxia. Despite the adipocyte-specific effects of individual SCFAs on the adipokine secretory profile, adipose tissue adipocytes are not the sole cellular source of various adipokines, in particular cytokines and chemokines, wherein immune cell populations that are either resident populations or recruited to obese adipose tissue are also capable of secreting these mediators [128,129]. Future studies should evaluate the effects of individual SCFAs on intact adipose tissue in both normoxic and hypoxic inflammatory conditions to capture the cellular cross-talk between adipocytes and immune cell populations that influence the secretory profile and metabolic function, which would increase the translational relevance of this work.

## 5. Conclusions

The adipocyte adipokine secretory profile that mechanistically connects adipose tissue inflammation to metabolic dysfunction can be dose-dependently attenuated by individual SCFAs, with the most potent effect attributable to butyrate, in combined inflammatory hypoxic environmental conditions that recapitulate the conditions of obese hypoxic adipose tissue [1,2,3,4,5,6,7,8,9,10,85]. The SCFA-mediated anti-inflammatory and anti-chemotactic effects on the adipokine secretory profile were most apparent at the higher 1 mM concentration, which is higher than typical circulating SCFA concentrations in humans [72,73,74,75,76]; however, this circulating concentration that would reach the adipose tissue could be achievable through encapsulated SCFAs [77]. Therefore, identifying both the individual SCFAs and optimal concentration that is most effective in attenuating adipocyte adipokine secretion could help to inform intervention strategies, via dietary prebiotic and/or SCFA-producing probiotic interventions, used alone or in combination with encapsulated SCFAs, to reach optimal circulating concentrations, which highlights the translational relevance of this work. Furthermore, this opens a new avenue of precision nutrition to provide individuals with optimal circulating levels of microbially derived metabolites typically produced from dietary precursors to support health, including adipose tissue function. Collectively, this work highlighting the effects of SCFAs on adipocyte function represents a potential intervention strategy to attenuate the severity of obese adipose tissue dysfunction by utilizing the gut–adipose tissue signaling pathway.

## Data Availability

The original contributions presented in this study are included in the article. Further inquiries can be directed to the corresponding author.
